# Comparative Biomechanical Evaluation of Novel Screwless Retained Dental Implant Prosthesis: A 3D Finite Element Analysis

**DOI:** 10.3390/jfb16020039

**Published:** 2025-01-22

**Authors:** Ki-Sun Lee, Jaeyeol Kim, JaeHyung Lim, Jae-Jun Ryu

**Affiliations:** 1 Department of Prosthodontics, Korea University Ansan Hospital, Ansan-si 15355, Republic of Korea; kisuns@gmail.com; 2 Medical Science Research Center, Korea University Ansan Hospital, Ansan-si 15355, Republic of Korea; 3 Department of Medicine, Graduate School, Korea University, Seoul 02841, Republic of Korea; jaeyeol.kim.kr@gmail.com; 4 Department of Oral & Maxillofacial Surgery, Korea University Ansan Hospital, Ansan-si 15355, Republic of Korea; 5 Department of Prosthodontics, Korea University Anam Hospital, Seoul 02841, Republic of Korea

**Keywords:** dental implant prosthesis, cementless screw-retained, finite element analysis

## Abstract

This study aimed to comparatively evaluate the biomechanical behaviors of three types of dental implant restorations: a screw-and-cement-retained prosthetic system (SCRP); a cementless screw-retained prosthetic system (SRP); and a novel screwless hook-retained prosthetic system (HRP). Three-dimensional finite element analysis (FEA) was used to evaluate biomechanical behavior. A comparative study of three dental implant prostheses was performed under two loading conditions: a vertical load of 100 N and an oblique load of 100 N at an angle of 30°. Under both loading conditions, the maximum von Mises stress values in the dental implant using the HRP system were lower (21.33 MPa) than those of the SCRP system (32.91 MPa), and the stress distribution of the implant prosthetic components tended to be more favorable than that of the SCRP system. Thus, the results show that the performance of the HRP system was comparable to that of a conventional SRP system under the same conditions. Regarding stress distribution, the novel screwless HRP system presents a viable alternative implant prosthodontic system to the conventional SCRP system.

## 1. Introduction

Dental implant treatment for teeth replacement has continuously improved over the past few years and represents a highly predictable therapy [1,2]. An important factor that ensures the success of dental implants is proper stress distribution around the fixture under a given masticatory load [3]. The stress distribution at the peripheral supporting bone around the implant may be variable depending on the applied load direction for restoration of the dental implant, supporting alveolar bone quality, dental implant fixture specifications [4,5,6], and implant–abutment–restoration component system [7,8]. All other things being equal, such as using the same implant fixture in the same bone and the same shape of the same material for upper crown restoration, the type of retaining method used in the abutment connecting the final restoration to the implant has a very different effect on load transfer [9]. The connection methods used between the abutment and crown restoration can typically be divided into screw-retained and cement-retained methods. Various screw-retaining and cement-retaining dental implant prosthodontic systems have been reported in the previous studies [10,11]. Screw-retained prostheses offer the advantage of retrievability, facilitating the detachment of the restoration from the fixture when required. They are particularly recommended in clinical scenarios where vertical space for restoration is limited to less than 4 mm. However, these prostheses are associated with certain disadvantages, including extended manufacturing time and costs, as well as the presence of a screw-access hole on the occlusal surface. In contrast, cement-retained restorations are characterized by a relatively straightforward fabrication process and a passive fit between the abutment and the dental implant fixture. Nonetheless, they present challenges such as difficulties in detaching the prostheses, potential biological risks stemming from residual cement, and unpredictable retention associated with the cementation process [11]. Consequently, the choice of retention method between the implant abutment and the dental prosthesis constitutes a critical clinical decision. It is important to recognize that bone is a dynamic living tissue that undergoes remodeling in response to various stimuli. Continuous overload on the peri-implant bone can result in bone resorption, which may ultimately lead to implant failure [6,12,13]. Therefore, a biomechanical evaluation of the implant fixture and the surrounding bone is essential for assessing the success of dental implants.

**Figure 1:**
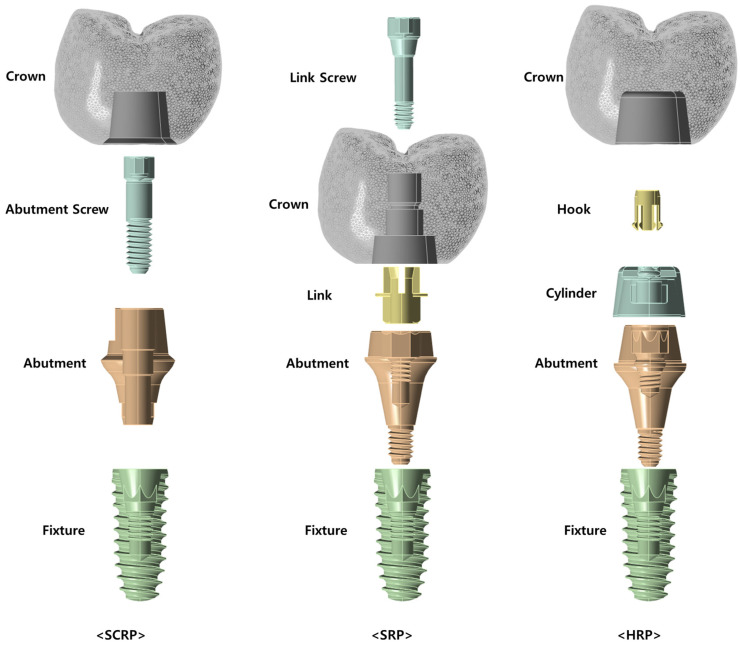
Conceptual diagram of the three dental implant prosthetic systems used in this study. SCRP: screw-and-cement-retained prosthetic system; SRP: screw-retained prosthetic system; HRP: hook-retained prosthetic system.

According to the previous studies for the distribution and concentration pattern of stress within the restoration-implant–bone complex, it can be influenced by the retention type of the implant-supported dental prostheses [14,15]. In conventional implant restorations, zirconia crowns are typically designed to be cement-retained, whereby they are affixed to a titanium abutment using dental cement (Figure 1—SCRP). Alternatively, a zirconia crown may be secured to a titanium link through a screw mechanism, referred to as the “screw-in-screw” technique. In this approach, a screw-type titanium base abutment is first attached to the implant, followed by the fixation of the titanium link and zirconia crown using a link screw (Figure 1—SRP). Recently, an innovative method has emerged that eliminates the need for both screws and cement, utilizing solely a hook to connect the abutment and crown restoration (Figure 1—HRP).

Figure 1 illustrates the configuration and assembly methods of the SCRP, SRP, and HRP systems based on 3D CAD designs. In the SCRP system, the crown is attached to the abutment using cement, and the abutment is connected to the fixture with a screw. In contrast, the SRP system employs a link and link screw to connect the crown and abutment, while the abutment itself is directly connected to the fixture without the need for additional screws. In the case of the HRP system, the crown is cemented to a component called a cylinder, which in turn is connected to the abutment via a hook component. Similar to the SRP system, the abutment of the HRP system is connected directly to the implant fixture without the need for an additional abutment screw.

The SRP system differs from the SCRP system in that it does not require an additional abutment screw to connect the abutment to the implant fixture. However, it does require a link and a link screw for the connection between the abutment and the crown, with the notable characteristic of not utilizing cement [9].

To date, no clinical trials or research papers have been published on the novel HRP system. the HRP system does not require an additional abutment screw to connect the abutment to the implant fixture, as is the case with the SRP system. However, unlike the SRP system, it requires a cylinder and a hook component for the connection between the abutment and the crown. Additionally, the HRP system does not use screws like the SRP system, and it requires the use of cement.

This novel dental implant prosthetic system addresses the limitations associated with the use of cement and screws in implant supported prosthetic systems. Accordingly, the present study assessed conventional screw and cement-retained zirconia crowns, cementless screw-retained zirconia crowns, and a novel design of and screwless hook-retained zirconia crowns using numerical analysis technique. The evaluation focused on the stress and strain experienced by the supporting implants and surrounding bone, employing three-dimensional (3D) finite element analysis (FEA).

## 2. Materials and Methods

### 2.1. 3D Model Design

**Figure 2:**
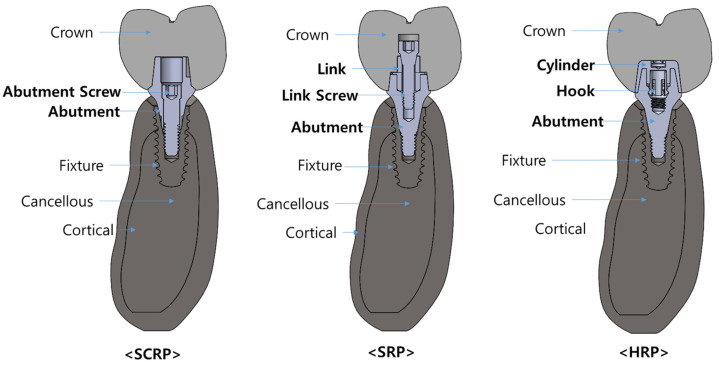
Conceptual diagram illustrating the combined models of SCRP, SRP, and HRP for each experimental group in this finite element analysis.

In this study, three different dental implant prosthetic systems were compared (Figure 2): single implants with a screw-and-cement-retained prosthetic system (SCRP), s cementless screw-retained prosthetic system (SRP), and screwless hook-retaining prosthetic (HRP) systems. To evaluate only the influence of the crown–implant connection type, the shapes and dimensions of the alveolar bone, implant fixture, and crown were assumed to be the same in all three 3D experimental models.

Three-dimensional alveolar bone model of lower second premolar region was designed using 3D modeling software ANSYS Workbench^®^ 2022 R2 (ANSYS Inc., Canonsburg, PA, USA) [16]. The implant fixture model used a CAD design of the IS-III fixture (NeoBiotech Co., Seoul, Republic of Korea) with a diameter of 4.0 mm and length of 10 mm (Ø4.0 × 10 mm).

The abutments, link, link screw, cylinder and hook 3D models used for SCRP, SRP, and HRP employed commercial products that are compatible with the IS-III implant fixture. Specifically, SCRP abutment employed SCRP multi abutment (Neobiotech Co., Seoul, Republic of Korea) with a specification Ø5.7 × 2mm, Higness Digital Link abutment (Highness Co., Ltd., Gyeongsangbuk-do, Republic of Korea) with a specification of Ø5.5 × 2 mm for SRP, and YK abutment (Neobiotech Co., Seoul, Republic of Korea) with a specification of Ø5.7 × 3 mm for HRP.

The 3D crown CAD design was created using a CAD program called Exocad (Exocad GmbH, Darmstadt, Germany) to create the same shape for all three experimental groups, based on the second premolar on the right side of the mandible with a buco-lingual width of 12 mm, a mesio-distal width of 10 mm and a height of 10 mm.

Three-dimensional CAD files of each implant and abutment component, as well as the design of the region inside the crown to which the abutment is connected to the crown, were provided by each vendor on the condition that they be used exclusively in this study.

### 2.2. Material Properties

All the materials used to construct the models were assumed to be isotropic, homogeneous, and linearly elastic. The mechanical properties of all the materials, such as the modulus of elasticity, Poisson’s ratio, and density, used in this study were obtained from a previous study [9] and also confirmed by the manufacturing company of each component (Table 1).

### 2.3. Loading Conditions

**Figure 3:**
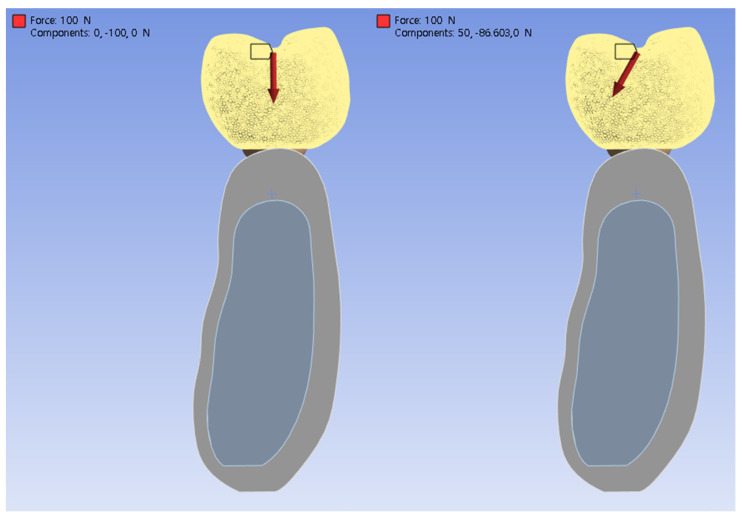
Conceptual illustration of the loading conditions: vertical (**left**) and inclined at 30° (**right**).

Two loading conditions were applied to each of the three experimental models. Referring to previous studies [6,9] that reported finite element analysis of the implant prosthesis, the occlusal force acting on the crown was set to 100 N, and the direction of the load was set perpendicular to the implant fixture and at an inclination angle of 30° (Figure 3). In this study, it was presumed that the contact condition between the implant and the bone was completely osseointegrated.

### 2.4. Finite Element Analysis

After all 3D models were imported into ANSYS software (ANSYS Inc., Canonsburg, PA, USA) and the mechanical properties were entered, all components of the model were meshed using parabolic tetrahedral elements under bonded surface contact conditions. After all three experimental models were simulated under the given two types of loading conditions, the maximum von Mises stress and strain values and distribution patterns at the surface of the implant and the adjacent bone structures were assessed. For quantitative analysis, stress distributions are illustrated through color-coded maps, where the lowest stress levels are denoted in blue and the highest in red.

## 3. Results

Table 2 and Table 3 list the maximum stress and strain values observed in the experimental models based on loading direction. Under both loading conditions, the maximum von Mises stress of the implant was greater in the SCRP-type than in the other cementless types (SRP) and screwless type (HRP). In the case of the SCRP model, the maximum stress on the implant was noticeably larger than that on the abutment components when comparing the SRP and HRP models, regardless of the loading conditions. The analysis revealed that both von Mises stress and strain in the surrounding bone were elevated during oblique loading compared to vertical loading, irrespective of the type of implant prosthesis used.

### 3.1. Bio-Mechanical Behaviors Under Vertical Loading Conditions

**Figure 4:**
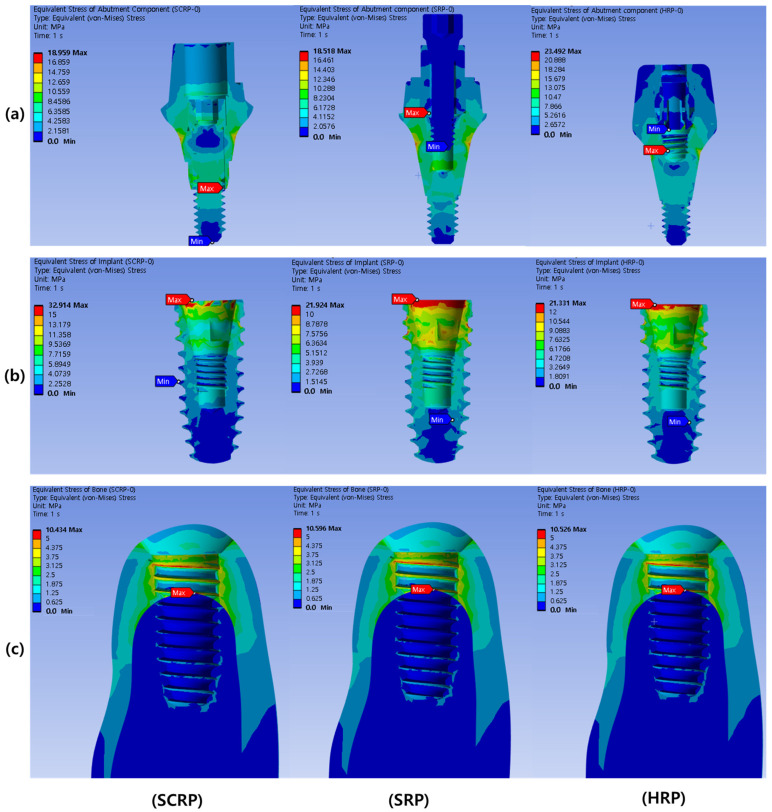
Color-coded von Mises stress distribution in abutment component under 100 N vertical load. The upper row represents the abutment components (**a**), the middle row represents the implant fixtures (**b**), and the lower row represents the surrounding bone of each experimental model (**c**).

Figure 4 shows the stress distribution in the implant abutment (a), implant fixture (b), and surrounding bone (c) when an axial load of 100 N was applied to each experimental model of SCRP, SRP, and HRP. The location where the maximum stress is concentrated in each experimental model is annotated with a red label. As shown in Figure 4a, the maximum stress concentration of the abutment components of all experimental models occurred inside the implant abutment. The maximum von Mises stress value in the abutment component of the HRP model (23.49 MPa) was larger than those of the SCRP (18.96 MPa) and SRP (18.52 MPa) (Table 2).

The maximum stress concentrations in the implant fixtures of all experimental models were observed at the crests of the implant fixtures (Figure 4). The maximum von Mises stress value in the implant of the SCRP model (32.91 MPa) was larger than that of the SRP (21.92 MPa) and HRP models (21.33 MPa) (Table 2).

In all the experimental models, the maximum stress concentration on the peri-implant bone was found around the implant neck near the area where the cortical and cancellous bones were in contact (Figure 4). The maximum von Mises stress values (range from 10.43 to 10.60 MPa) and maximum strain values (range from 0.00108 to 0.00110 mm/mm) in the peri-implant bone of all experimental models were similar regardless of prosthetic types (Table 2).

### 3.2. Bio-Mechanical Behaviors Under Oblique Loading Conditions

**Figure 5:**
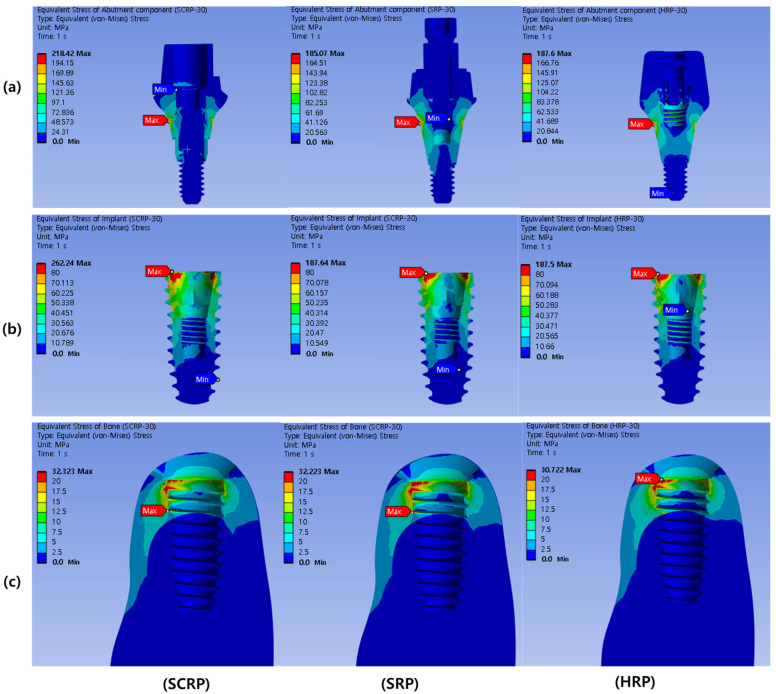
Color-coded von Mises stress distribution in abutment component under 100 N oblique load (30°). The upper row represents the abutment components (**a**), the middle row represents the implant fixtures (**b**), and the lower row represents the surrounding bone of each experimental model (**c**).

Figure 5 shows the stress distribution in the implant abutment (a), implant fixture (b), and bone surrounding the implant (c) when an oblique load (30°) of 100 N was applied to each experimental model of SCRP, SRP, and HRP. The location where the maximum stress is concentrated in each experimental model is annotated with a red label. As shown in Figure 5a, the maximum stress concentration of the abutment components of all experimental models occurred outside the implant abutment. The maximum von Mises stress value in the abutment component of the SCRP model (218.42 MPa) was larger than that of the SRP (185.07 MPa) and HRP (187.60 MPa) (Table 3).

The maximum stress concentrations in the implant fixtures of all experimental models were observed at the crests of the implant fixtures (Figure 5). The maximum von Mises stress value in the implant of the SCRP model (262.24 MPa) was greater than those of the SRP (187.64 MPa) and HRP (187.50 MPa) (Table 3).

In all the experimental models, the maximum stress concentration on the peri-implant bone was found around the implant neck, near the area where the cortical and cancellous bones were in contact (Figure 5). The maximum von Mises stress values (32.22–32.72 MPa) and maximum strain values (0.00235–0.00238 mm/mm) in the peri-implant bone of all experimental models were similar regardless of prosthetic types (Table 2).

## 4. Discussion

The results of this study showed that the type of retaining method used in the implant prosthesis significantly affected the stress distribution in the implant fixture. This FEA study presented a novel cement and screwless-retained crown-designed HRP system, exhibiting favorable strain and stress distribution compared to the model with a conventional SCRP, while also showing equivalent performance to a conventional cementless SRP.

In terms of the masticatory force used in the finite element analysis of dental implants, previous studies have shown that it can be performed under various conditions such as the masticatory force value and direction, static or dynamic settings [5]. The magnitude acting on individual teeth depending on human masticatory activity varies greatly, it has been studied to be between 20 and 120 N on average [17,18]. Hence, this study adopted a static load value of 100 N, which is commonly used in finite element analysis of dental implants [6,19,20,21], and for comparison with similar studies on load distribution according to abutment type [9].

In this study, the cementless screw-retained restoration system (SRP) and screwless hook-retained restoration system (HRP) contained one more component than the SCRP system (Figure 1). The SRP has three abutment components: the abutment, link, and link screw. The HRP also has three abutment components: the abutment, hook, and cylinder. However, SCRP has only two abutment components, the abutment and abutment screw, which explains why the stress values on the implant were lower in the SRP and HRP models than in the SCRP model and why the stress distribution between the abutment components and implant fixture in both the models was more favorable.

The above results are accordance with those of previous studies [9,22] that suggested that more parts consisting of an abutment component show more favorable biomechanical behaviors. These studies found that greater stress values might be detected in the abutment components (abutments and screws) before the stress reaches the bone–implant contact area. As explained in a previous report, the tolerance of implant components can reduce the stress transmitted to the surrounding bone depending on the components moving freely [23].

The conventional SCRP prosthetic system provides the advantage of reducing the number of screw utilization and minimizing micromovement of the prosthesis. As a result, this reduction can contribute to a decreased risk of screw loosening and associated prosthetic complications. However, it may also present challenges in alleviating the transmission of masticatory forces to the implant and the surrounding osseous structures. Specifically, the forces exerted on the occlusal surface may be transmitted directly to the bone, rather than being absorbed by the prosthetic components. As a result, these mechanical dynamics could potentially elevate stress levels on the peri-implant hard tissue.

In all experimental models, the maximum von Mises stress of the implant was observed at the crest of the implant fixture, corroborating findings from prior studies [24]. When the stress transferred to the alveolar crest exceeds the bone’s elastic limit, it heightens the risk of alveolar bone fracture and can result in marginal bone loss [25]. To prevent this complication, it is essential to ensure favorable bone quality around the crestal area of the implant [26].

In the present study, the stress associated with the cementless screw-retained restoration (SRP) was distributed across the implant and prosthetic components, rather than being localized at the implant neck. The highest stress concentration was identified in the crestal region of the implant under both axial and oblique loads (refer to Table 2 and Table 3 and Figure 4 and Figure 5). This suggests that the stress distribution of cementless restoration system (SRP) and screwless restoration system (HRP) with multiple components are more advantageous compared to that of the conventional SCRP system.

Furthermore, this study revealed a greater concentration of stress under oblique loading compared to vertical loading, irrespective of the type of dental implant prosthetic utilized. These findings align with those of previous research [23,27]. Consequently, positioning the implant fixture in alignment with the direction of masticatory load and designing a crown with a reduced cusp slope are beneficial strategies for minimizing oblique overload and decreasing deformation.

This study utilized finite element analysis (FEA) to simulate three experimental groups. As FEA is a numerical analysis-based method, it yields consistent results regardless of repeated simulations. Therefore, there is a limitation that statistical processing to obtain statistical significance cannot be performed such as ANOVA tests. Due to this limitation, it is not possible to establish or evaluate a null hypothesis. Instead, the findings are interpreted based on quantitative and qualitative results.

FEA provided a numerical representation of the distribution of stress and strain values across different types of implant prostheses. However, due to the inherent limitations of FEA technique, utilized in this research do not precisely represent the true oral conditions in mankind. Variations in the shapes and mechanical properties of cortical and cancellous bone exist among individuals. Therefore, additional clinical studies or more complicated FEA studies that take these differences into account are necessary to validate the results of the current investigation, such as considering the complex anisotropic properties of real bone and the cyclic loading to reflect human masticatory activity.

## 5. Conclusions

This FEA study revealed that the novel screwless hook retaining abutment system (HRP) resulted in lower stress concentration on the implant compared to the conventional screw- and cement-retained prosthesis system (SCRP). Additionally, the HRP system demonstrated comparable performance to the cementless screw-retained prosthesis system (SRP), suggesting it may serve as a favorably alternative to traditional screw- and cement-retained prosthetic systems.

## Figures and Tables

**Table 1:** Mechanical properties of materials used in this study.

Component	Modulus of Elasticity (GPa)	Poisson’s Ratio (ν)	Density (kg/m^3^)
Cancellous Bone	1.3	0.30	500
Cortical Bone	13	0.30	1180
Titanium (Ti-Gr4) (Fixture)	103	0.33	4620
Titanium (Ti-6Al-4V) (Abutment, Screw, Link, Link Screw, Cylinder, Hook)	103	0.33	4620
Zirconia	200	0.31	6090

**Table 2:** Maximum von Mises stress values in each component of experimental models and maximum strain value in alveolar bone under vertical load of 100 N.

Components	SCRP	SRP	HRP
Abutment Component (Stress)	18.96 MPa	18.52 MPa	23.49 MPa
Implant (stress)	32.91 MPa	21.92 MPa	21.33 MPa
Bone (stress)	10.43 MPa	10.60 MPa	10.54 MPa
Bone (strain)	0.00108 mm/mm	0.00110 mm/mm	0.00109 mm/mm

**Table 3:** Maximum von Mises stress values in each component of experimental models and maximum strain value in alveolar bone under 30° oblique load of 100 N.

Components	SCRP	SRP	HRP
Abutment Component (Stress)	218.42 MPa	185.07 MPa	187.60 MPa
Implant (stress)	262.24 MPa	187.64 MPa	187.50 MPa
Bone (stress)	32.32 MPa	32.22 MPa	30.72 MPa
Bone (strain)	0.00238	0.00237	0.00235

## Data Availability

The datasets presented in this article are not readily available because the CAD data used for analysis was provided by each company as the blueprint of the product under the condition that it would be used only for finite element analysis in this study and that it would not be shared with external organizations or individuals. Requests to access the datasets should be directed to Ki-Sun Lee (kisuns@gmail.com).
